# Effect of low-cost direct ophthalmoscopy training on glaucoma disc assessment by allied ophthalmic personnel students: a controlled pre-post study

**DOI:** 10.1038/s41433-026-04477-2

**Published:** 2026-05-06

**Authors:** Vrundaben Patel, Andreas Katsimpris, John Buchan, Kangwa Muma, Andrew Blaikie

**Affiliations:** 1 University Teaching Hospitals -Eye Hospital, Lusaka, Zambia; 2 NHS Fife, Scotland, UK; 3 International Centre for Eye Health, London School of Hygiene and Tropical Medicine, London, UK; 4 University of Lusaka, Lusaka, Zambia; 5 University of St Andrews, Fife, Scotland, UK

**Keywords:** Physical examination, Optic nerve diseases

Early detection and treatment of glaucoma can help prevent progression to irreversible blindness, yet in many countries the majority affected remain undiagnosed [1]. With only one ophthalmologist per 556 000 population in Zambia as of 2022 [2], the burden of early glaucoma detection falls on Allied Ophthalmic Personnel (AOP) working at the district and provincial health service level.

This study aimed to evaluate the impact of a single day of focussed direct ophthalmoscopy training using a frugal solar-powered direct ophthalmoscope and low-cost ophthalmoscopy simulation eyes on ophthalmoscopy skills, disc assessment, and knowledge about glaucoma amongst undergraduate AOPs in Zambia.

Thirty final-year AOP students [Medical Licentiate- Ophthalmology (Bachelor of Science (BSc) in Clinical Ophthalmology] and ophthalmic nurses (BSc in Ophthalmic Nursing)] at Levy Mwanawasa Medical University were randomised into an intervention group (*n* = 15) [who underwent a one-day training, with ophthalmoscopy simulation eyes and the Arclight direct ophthalmoscope, and retained the ophthalmoscope], or a control group (*n* = 15), who engaged in unguided ophthalmoscopy practice and returned the device (Fig. 1).

**Fig. 1:**
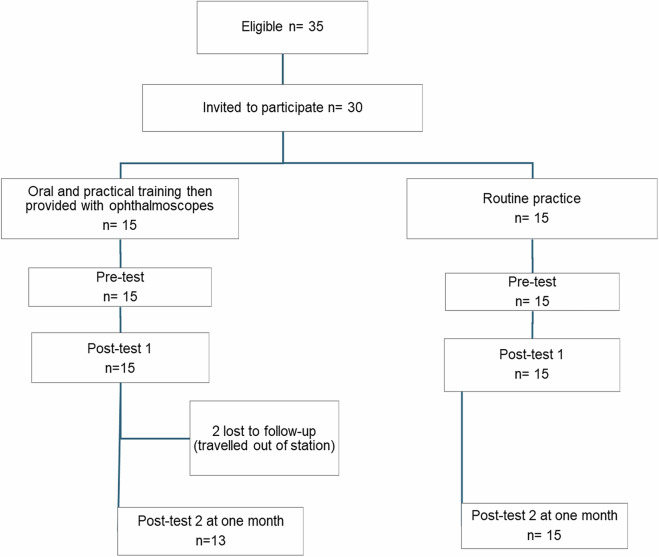
Participant recruitment flow-chart. This diagram shows the enrolment and assignment of participants to the intervention (training and provision of Arclight ophthalmoscopes) and control (routing practice) groups from baseline to final follow-up at one month.

Knowledge, direct-ophthalmoscopy (DO) technique, glaucoma feature-recognition, and disc-classification skills were evaluated at baseline, immediately post-training, and one month later. The primary outcome was change in feature-recognition score from baseline to one month. Participants were also scored on patient disc examination at one month.

Baseline scores were similar between groups. Both groups demonstrated significant gains in knowledge and direct ophthalmoscopy skills over the study period, indicating that structured exposure and repeated assessment alone can enhance learning in AOP students. The intervention group (*n* = 13), compared to the control group (*n* = 15), demonstrated greater improvements in all domains except one. These included overall knowledge ( + 24.5% vs +11.6%, *p* = 0.03), examination technique ( + 16.5% vs +0.3%, *p* = 0.046) and disc classification ( + 16.2% vs +9.2%, *p* = 0.027). However, improvement in recognition of specific glaucomatous optic disc features was similar in both groups ( + 13.4% vs +12.0%, *p* = 0.673)- Fig. 2. The limited prior ophthalmoscopy experience (80% had performed direct ophthalmoscopy less than 5 times) reported by all participants, despite being at the end of their training, was noteworthy. Importantly, the satisfactory performance on real patient examinations at one month suggests that the improvements observed in both groups translated into clinically relevant skills.

**Fig. 2:**
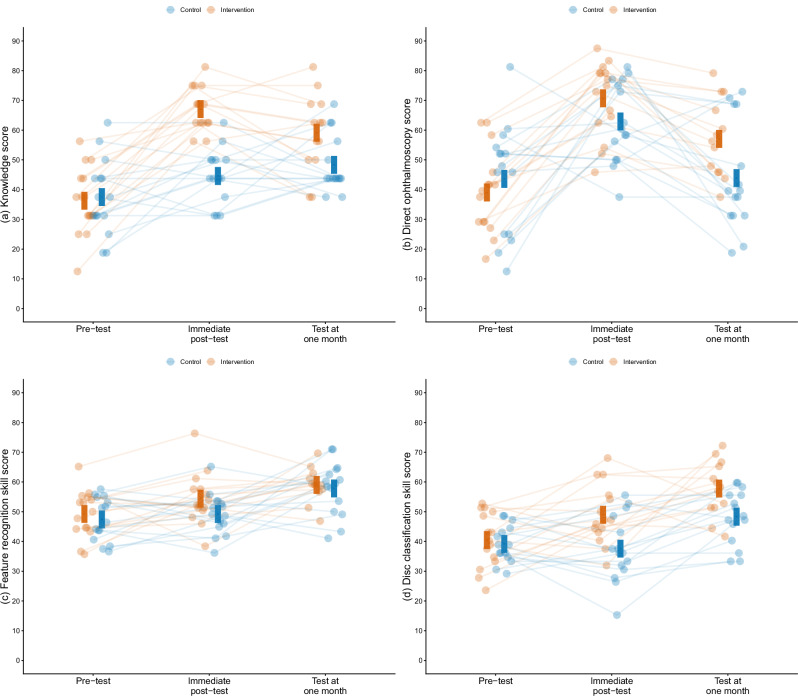
Distribution of knowledge and skills scores for the three assessments. This is a compilation of trend in scores for the intervention (orange) and control (blue) group participants across all fields assessed. Part (**a**) shows the comparison in knowledge, part (**b**) shows the results for direct ophthalmoscopy skill scores, part (**c**) shows the glaucoma feature-recognition on optic disc examination skill scores, and part (**d**) compares scores for skill in classifying optic discs as normal vs abnormal non-glaucomatous vs abnormal glaucomatous.

This study combined targeted training with use of low cost diagnostic and training tools to improve knowledge and skills of health workers as previous research suggested only using one without the other may limit sustained improvement [3, 4].

Targeted one-day instruction using the frugal Arclight ophthalmoscope and glaucoma simulation eyes produced meaningful, sustained gains in direct-ophthalmoscopy knowledge and disc-classification accuracy among undergraduate AOP. Modest gains in recognising specific glaucomatous features highlight the need for ongoing reinforcement, such as remote case-based mentoring or periodic refresher training as recommended in the Sub-Saharan Africa Glaucoma Toolkit [5]. Embedding frugal, competency-based modules in AOP undergraduate curricula can expand and strengthen glaucoma detection reducing preventable blindness in similar high-burden settings

## Dataset

The datasets generated and analysed during this study are available from the corresponding author on reasonable request.

## References

[1] Damji KF, Nazarali S, Giorgis A, Kiage D, Marco S, Philippin H, et al. STOP Glaucoma in Sub Saharan Africa: enhancing awareness, detection, management, and capacity for glaucoma care. Expert Rev Ophthalmol [Internet]. 2017;12:197–206. [cited 2023 Dec 6] Available from https://www.tandfonline.com/doi/full/10.1080/17469899.2017.1295848.

[2] Ministry of Health. National Health Strategic Plan (NHSP) 2022–2026. Government of Zambia; 2023.

[3] Gilmour-White JA, Picton A, Blaikie A, Denniston AK, Blanch R, Coleman J, et al. Does access to a portable ophthalmoscope improve skill acquisition in direct ophthalmoscopy? A method comparison study in undergraduate medical education. BMC Med Educ [Internet]. 2019;19:201 [cited 2024 Jan 14] Available from: https://bmcmededuc.biomedcentral.com/articles/10.1186/s12909-019-1644-5. 31196068 10.1186/s12909-019-1644-5 PMC6567496

[4] Malik ANJ, Mafwiri M, Gilbert C, Kim MJ, Schellenberg J. Integrating eye health training into the primary child healthcare programme in Tanzania: a pre-training and post-training study. BMJ Paediatr Open. 2020;4:e000629 [cited 2024 Jan 30]Available from https://bmjpaedsopen.bmj.com/lookup/doi/10.1136/bmjpo-2019-000629. 32671232 10.1136/bmjpo-2019-000629 PMC7351275

[5] Kyari F, Abdull M, Choudhari N, Kiage D, Karinya L, Ogundimu K, et al. A Toolkit for Glaucoma Management in Sub-Saharan Africa. International Agency for the Prevention of Blindness; 2021.

